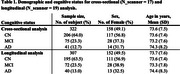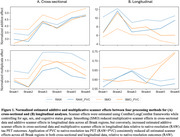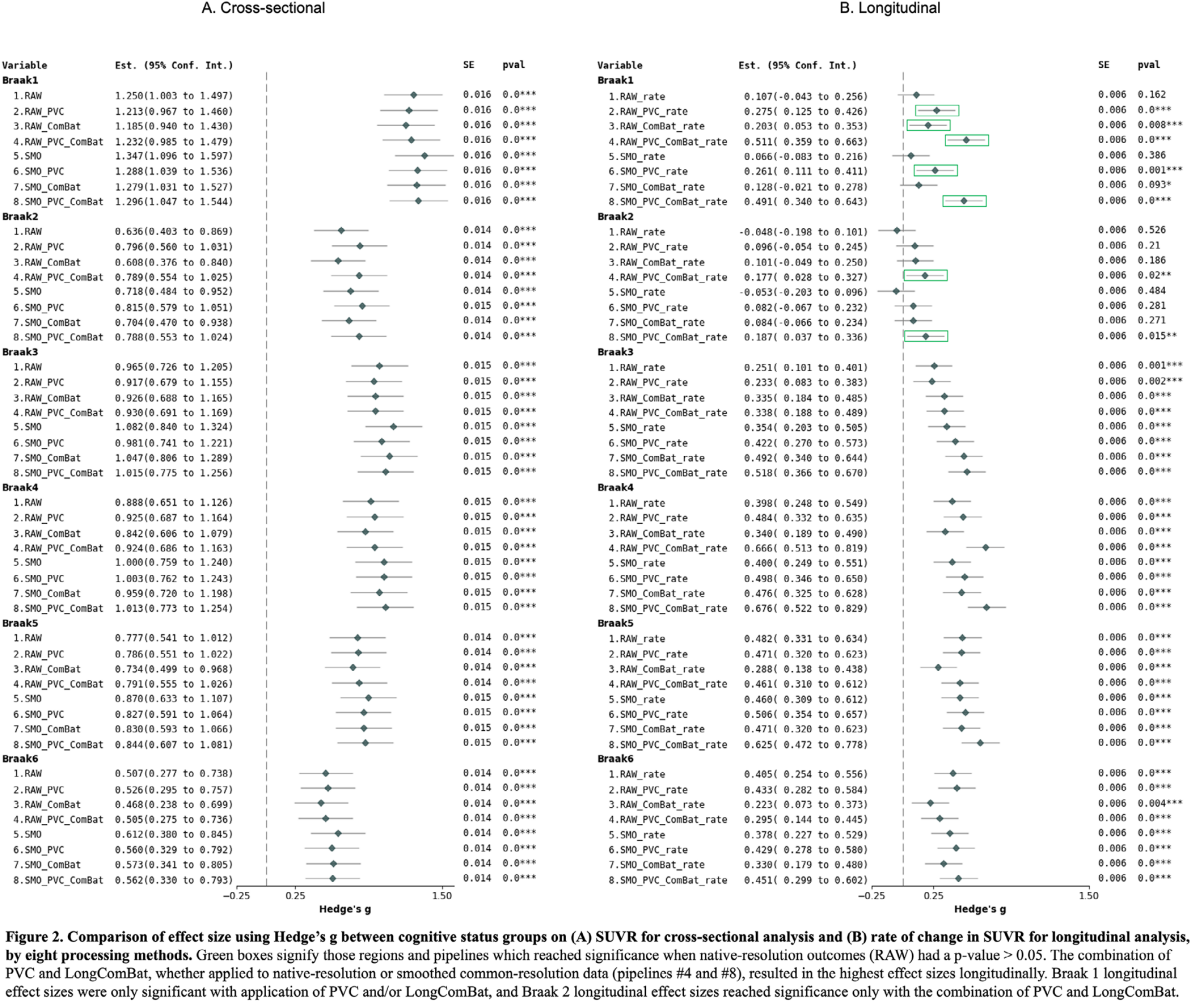# Effect of image‐processing and statistical harmonization methods on tau PET cognitive group separability for multisite cross‐sectional and longitudinal studies using ^18^F‐Flortaucipir PET

**DOI:** 10.1002/alz70856_107440

**Published:** 2026-01-09

**Authors:** Weiquan Luo, Charles M Laymon, Suzanne L. Baker, Howard J Aizenstein, Dana L Tudorascu, Davneet S Minhas, Alzheimer's Disease Neuroimaging Initiative

**Affiliations:** ^1^ University of Pittsburgh, Pittsburgh, PA, USA; ^2^ Lawrence Berkeley National Laboratory, Berkeley, CA, USA

## Abstract

**Background:**

Gaussian smoothing to a common resolution is regularly employed to harmonize PET imaging data acquired across different scanners in multisite studies. However, spatial smoothing of PET can increase spill‐over contamination between neighboring regions. Partial volume correction (PVC) has, in turn, been applied to correct for such contamination. Despite being common practices, the harmonizing impact of smoothing and PVC on tau PET data remains unclear.

Evaluate the impact of eight image‐processing and statistical harmonization pipelines on estimated scanner (batch) effects and cognitive status group separation in cross‐sectional and longitudinal analyses of multisite ^18^F‐Flortaucipir (FTP) PET data.

**Method:**

Native‐resolution FTP PET images (RAW) from 322 ADNI participants scanned at up to 4 different time points on 19 different PET scanners were included in the analyses (Table 1). Putative image‐processing harmonization pipelines evaluated were the combination of the three processing steps: (1) smoothing of PET images to an effective resolution of 8mm (SMO); (2) application of a tau‐specific PVC method (Baker et al, 2017; PVC); and (3) application of ComBat/LongComBat harmonization methods to cross‐sectional/longitudinal regional values (ComBat).

SUVR outcomes were extracted for all methods from Braak regions. Mean normalized additive and multiplicative cross‐sectional and longitudinal scanner effects across sites were assessed for image‐processing pipelines using ComBat and LongComBat frameworks. Hedge's *g* effect sizes were evaluated for all pipelines between cognitive status groups (cognitively normal [CN] versus non‐normal [MCI&AD]) cross‐sectionally on regional SUVR and longitudinally on yearly rate of SUVR change.

**Result:**

Smoothing did not consistently reduce estimated scanner effects, cross‐sectionally or longitudinally (Figure 1). However, PVC of native‐resolution tau PET data (RAW+PVC) consistently reduced estimated scanner effects across all Braak regions, relative to RAW. The combination of PVC and LongComBat harmonization, whether applied to native‐resolution (RAW) or common‐resolution (SMO) PET images, resulted in the greatest longitudinal effect sizes across all Braak regions (Figure 2).

**Conclusion:**

PVC of tau PET data may have a greater harmonizing effect on multi‐scanner tau PET data than smoothing to a common resolution. The combination of PVC with LongComBat maximizes the ability to detect group differences in longitudinal change of multi‐site FTP PET data.